# Tamoxifen's protection against breast cancer recurrence is not reduced by concurrent use of the SSRI citalopram

**DOI:** 10.1038/sj.bjc.6604533

**Published:** 2008-07-29

**Authors:** T L Lash, L Pedersen, D Cronin-Fenton, T P Ahern, C L Rosenberg, K L Lunetta, R A Silliman, S Hamilton-Dutoit, J P Garne, M Ewertz, H T Sørensen

**Affiliations:** 1 Department of Epidemiology, Boston University School of Public Health, 715 Albany Street, Boston, MA 02118, USA; 2 Department of Clinical Epidemiology, Aarhus University Hospital, Olof Palmes Alle 43, Aarhus N 8200, Denmark; 3 Department of Medicine, Boston University School of Medicine, 715 Albany Street, Boston, MA 02118, USA; 4 Department of Biostatistics, Boston University School of Public Health, 715 Albany Street, Boston, MA 02118, USA; 5 Institute of Pathology, Aarhus University, Noerrebrogade 44, Aarhus C 8000, Denmark; 6 Department of Surgery, Aalborg Hospital, Aarhus University Hospital, Hobrovej 18-22, Aalborg 9100, Denmark; 7 On behalf of the Danish Breast Cancer Co-operative Group (DBCG); 8 Department of Oncology, Odense University Hospital, Sdr. Boulevard 29, Odense C 5000, Denmark

**Keywords:** breast neoplasms, pharmacology and therapeutic use, tamoxifen, antagonists and inhibitors, serotonin reuptake inhibitors, cytochrome P-450 2D6

## Abstract

Tamoxifen remains an important adjuvant therapy to reduce the rate of breast cancer recurrence among patients with oestrogen-receptor-positive tumours. Cytochrome P-450 2D6 metabolises tamoxifen to metabolites that more readily bind the oestrogen receptor. This enzyme also metabolises selective serotonin reuptake inhibitors (SSRI), so these widely used drugs – when taken concurrently – may reduce tamoxifen's prevention of breast cancer recurrence. We studied citalopram use in 184 cases of breast cancer recurrence and 184 matched controls without recurrence after equivalent follow-up. Cases and controls were nested in a population of female residents of Northern Denmark with stages I–III oestrogen-receptor-positive breast cancer 1985–2001 and who took tamoxifen for 1, 2, or most often for 5 years. We ascertained prescription histories by linking participants' central personal registry numbers to prescription databases from the National Health Service. Seventeen cases (9%) and 21 controls (11%) received at least one prescription for the SSRI citalopram while taking tamoxifen (adjusted conditional odds ratio=0.85, 95% confidence interval=0.42, 1.7). We also observed no reduction of tamoxifen effectiveness among regular citalopram users (⩾30% overlap with tamoxifen use). These results suggest that concurrent use of citalopram does not reduce tamoxifen's prevention of breast cancer recurrence.

Tamoxifen is a selective oestrogen receptor modulator (Jordan and Dowse, 1976) that reduces by half the risk of breast cancer recurrence in early-stage patients whose tumour cells express the oestrogen receptor (Early Breast Cancer Trialists' Collaborative Group, 2005). To be pharmacologically active, tamoxifen must be metabolised to secondary metabolites that bind the oestrogen receptor 100-fold more readily than tamoxifen itself (Malet *et al*, 1988). Four cytochrome P-450 enzymes (CYPs) catalyse this activation (CYP2D6, CYP3A4, CYP3A5, and CYP2C9) (Malet *et al*, 1988). CYP2D6 catalyses formation of 4-hydroxytamoxifen from tamoxifen (Coller *et al*, 2002) and formation of 4-hydroxy-*N*-desmethyltamoxifen from *N*-desmethyltamoxifen (Stearns *et al*, 2003). These two secondary metabolites have the highest binding affinity for the oestrogen receptor, and binding affinity correlates with inhibition of cell growth (Coezy *et al*, 1982). The secondary metabolites are, therefore, the most important modulators of the oestrogen receptor in the tamoxifen pathway (Lim *et al*, 2005).

Breast cancer patients treated with tamoxifen may also take other prescription medications that are metabolised by some of the same enzymes that activate tamoxifen. For example, depression is a common comorbidity in breast cancer patients (Massie, 2004), and many selective serotonin reuptake inhibitors (SSRI), which are widely used medications indicated primarily to treat depression (Hansen *et al*, 2003), are metabolised by CYP2D6 (Zanger *et al*, 2004). SSRI competition with tamoxifen and *N*-desmethyltamoxifen for CYP2D6, or direct inhibition of CYP2D6 by SSRI, could reduce the production of the tamoxifen metabolites with high receptor-binding affinity, and thereby reduce tamoxifen's prevention of breast cancer recurrence. Competition between tamoxifen and the SSRI paroxetine reduced the plasma concentration of endoxifen in a cross-over clinical trial (Stearns *et al*, 2003). Furthermore, the mean plasma concentration of 4-hydroxy-*N*-desmethyltamoxifen was more than two-fold greater among women who were taking no CYP2D6 competitor drug than among women who were taking such a drug (Jin *et al*, 2005). *In vivo* studies thus demonstrate a compelling biological basis for the hypothesis that concomitant use of SSRI would reduce tamoxifen's prevention of breast cancer recurrence.

In the largest study to date of the potential for drug–drug interaction to reduce tamoxifen's protection against breast cancer recurrence, we examined whether Danish breast cancer patients with oestrogen-receptor-positive tumours who were treated with tamoxifen for 1, 2, or most often for 5 years had a higher rate of recurrence if they were concomitantly taking the SSRI citalopram or its S-stereoisomer (‘citalopram’ from here onwards) than if they were not. As described in more detail below, citalopram was the most frequently prescribed SSRI in the study population.

## Materials and methods

The study was approved by the Boston University Medical Campus Institutional Review Board and The Regional Committee on Biomedical Research Ethics of Aarhus County.

### Study population

The source population included female residents of four Northern Danish counties (Aarhus, North Jutland, Viborg, and Ringkøbing) aged 35–69 at diagnosis of primary International Union Against Cancer stage I, II, or III breast cancer (UICC, 1997) between 1985 and 2001 and who were reported to the Danish Breast Cancer Cooperative Group (DBCG). The DBCG has enrolled nearly all Danish breast cancer patients younger than age 70 at diagnosis into its clinical database since 1977 (Andersen and Mouridsen, 1988; Jensen *et al*, 2003). More than 90% of Danish breast cancer cases are reported to the DBCG and more than half of the DBCG patients are enrolled in clinical trials (Andersen and Mouridsen, 1988). The same standardized forms are used to follow all patients reported to the DBCG, regardless of whether they enrol in a trial, so the registry provides the data quality advantage of a clinical trial setting with the generalisability advantage of a population-based setting.

We divided the source population into three groups: (a) group I women whose tumour expressed the oestrogen receptor protein and who were treated with tamoxifen for at least 1 year; (b) group II women whose tumour did not express the oestrogen receptor protein, were not treated with tamoxifen, and who survived for at least one year; and (c) group III women, comprising all others, who were excluded from this analysis. Group I women were assigned to tamoxifen therapy protocols of 1, 2, or 5 years, depending on the guideline extant in Denmark at the time of their diagnoses. We included group II women to estimate the direct association of citalopram prescription with recurrence rate, if any. We further restricted the source population to women diagnosed with breast cancer after the date that their county of residence began to maintain an electronic prescription database (Aarhus=1996, North Jutland=1989, Ringkøbing=1998, Viborg=1998), which were used to ascertain use of prescription medications, including citalopram. Follow-up time began 1 year after the date of breast cancer diagnosis and continued until the date of the first of breast cancer recurrence, death from any cause, loss to follow-up (e.g., emigration), 10 years of follow-up, or 1 September 2006.

Cases were women with local or distant breast cancer recurrence occurring during their follow-up time among the members of groups I and II. We selected one control for each case without replacement from members of the source population who had not had a breast cancer recurrence after the same amount of follow-up time. We matched controls to cases on (a) group membership (group I or II), (b) menopausal status at diagnosis (premenopausal or postmenopausal), (c) date of breast cancer surgery (caliper matched±12 months), (d) county of residence at the time of diagnosis, and (e) UICC stage at diagnosis (stage I, II, or III).

### Data collection

We used the Danish Civil Personal Registration (CPR) number assigned to each case and control to link data sets. The CPR is a unique identification number assigned to all Danish residents alive on 1 April 1968, born thereafter, or upon immigration.

We collected demographic information (age, menopausal status, and hospital of diagnosis), tumour characteristics (UICC stage, histological grade, and oestrogen-receptor expression), and therapy characteristics (primary surgical tumour management, receipt of radiation therapy, receipt of chemotherapy, and receipt of tamoxifen therapy) from the DBCG database.

We collected data on receipt of citalopram prescription and other potential CYP2D6 inhibitors (including other SSRI) by linking the CPR number of cases and controls to the prescription databases maintained by each county (see, for example, the description of North Jutland's database (Gaist *et al*, 1997)).

### Analytic variables

#### Recurrence

We used the DBCG definition of breast cancer recurrence as any type of breast cancer subsequent to the initial course of therapy (Andersen and Mouridsen, 1988). Given the definition of the source population and follow-up time, all cases of recurrence occurred between 1 and 10 years after the primary breast cancer diagnosis.

#### Prescription status

Prescription medications are coded by the Anatomical Therapeutic Chemical (ATC) classification system (WHO Collaborating Centre for Drug Statistics Methodology, 2007). We defined SSRI antidepressants as all those classified in group N06AB by the ATC. These are the SSRI drugs: zimeldine (N06AB02), fluoxetine (N06AB03), citalopram (N06AB04), paroxetine (N06AB05), sertraline (N06AB06), alaproclate (N06AB07), fluvoxamine (N06AB08), etoperidone (N06AB09), and escitalopram (N06AB10). We defined citalopram exposure as any prescription for citalopram (N06AB04) or its S-stereoisomer escitalopram (N06AB10).

We classified cases and controls as those with no record of a citalopram prescription during their follow-up time (never citalopram) and those with any record of prescription for citalopram during their follow-up time (ever citalopram). We used a similar procedure to classify cases and controls as ever or never users of another SSRI or of another prescription medication that is a CYP2D6 inhibitor or substrate, aside from those indicated to treat breast cancer recurrence or its effects. See the Supplementary online material for a complete list of these medications and the frequency of their use in the study population.

For group I women who ever had a citalopram prescription, we calculated the percentage of time on tamoxifen when they were simultaneously taking citalopram. We created categories of (a) intermittent citalopram use, defined as citalopram use overlapping tamoxifen use for more than 0% but less than 30% of the time on tamoxifen and (b) regular citalopram use, defined as citalopram use overlapping tamoxifen use for 30% or more of the time on tamoxifen. We chose 30% as the overlap boundary to allow sufficient sample size in the regular citalopram subgroup, while also investigating a substantial period of SSRI and tamoxifen comedication.

#### Covariates

We defined the following set of covariates: (a) time period of breast cancer diagnosis (1985–1993, 1994–1996, and 1997–2001), (b) age at diagnosis (35–44 years, 45–54 years, 55–64 years, and 65–70 years), (c) menopausal status at diagnosis (premenopausal and postmenopausal), (d) county of residence at diagnosis (Aarhus, North Jutland, Viborg, and Ringkøbing), (e) UICC stage at diagnosis (stages I, II, and III), histological grade (grade I, II, III, and missing), surgery type (breast conserving surgery and mastectomy), and receipt of systemic adjuvant chemotherapy (yes and no), and (f) receipt of a prescription for another medication that is a CYP2D6 inhibitor or substrate, including other SSRI, while taking tamoxifen.

### Analytic strategy

All analyses were conducted within strata of the two groups (oestrogen-receptor positive and treated with tamoxifen or oestrogen-receptor negative and not treated with tamoxifen). We computed the frequency and proportion of cases and controls within categories of assigned protocol of tamoxifen duration, of citalopram use, of use of other CYP2D6 inhibitors or substrates, and of the covariates. We calculated the number of cases and controls ever receiving citalopram, the number of total prescriptions for citalopram summed over all cases or controls, and the range of the number of prescriptions for citalopram received by each individual case or control.

We estimated the rate ratio associating citalopram prescription with breast cancer recurrence as the odds ratio (OR) in a conditional logistic regression including only citalopram use as the exposure variable and conditioned on the matched factors. By design, this ratio adjusts for confounding by the matched factors (Greenland, 2008). We examined whether the effect of citalopram use was modified by duration of tamoxifen therapy in a stratified analysis. Finally, we adjusted for residual confounding by the covariates that were not included in the matching by including them as independent variables in the conditional logistic regression. We retained in the final model any covariate that affected the log OR from the conditional logistic regression model associating citalopram use with breast cancer recurrence rate by more than 10% (Greenland, 1989). All estimates are accompanied by a 95% confidence interval (CI) calculated by the profile likelihood method. All analyses were performed using SAS version 9.

## Results

Table 1 shows the frequency and proportion of cases and controls, within strata of group, in the categories of the covariates. About two-thirds of cases and controls in both groups were diagnosed with primary breast cancer during the period 1997–2001, and the majority was resident in Aarhus or North Jutland counties, because the prescription registries began first in these two counties. A large majority had mastectomy as their primary surgical intervention, which is consistent with the clinical practice pattern previously reported in this region during this time period (Ahern *et al*, 2008). Group I women (positive oestrogen-receptor expression and treated with tamoxifen) were more likely to be post-menopausal (87%) than were group II women (66%; negative oestrogen-receptor expression and not treated with tamoxifen). Group I women were also less likely to receive systemic adjuvant chemotherapy (11 and 13% of cases and controls, respectively) than were group II women (80 and 70% of cases and controls, respectively); reflecting the preference for hormonal therapy over systemic adjuvant chemotherapy in women whose tumours expressed the oestrogen receptor. Between 3 and 11% of cases and controls ever used citalopram while taking tamoxifen (group I) or during their follow-up period (group II).

Table 2 depicts the pattern of SSRI prescriptions received by cases and controls. In both groups, SSRI prescriptions were primarily written for citalopram or its S-stereoisomer, escitalopram. For example, 17 of 23 group I cases (74%) ever prescribed an SSRI had at least one prescription for citalopram, accounting for 86% of the total number of prescriptions. Similarly, 22 of 30 group I controls (73%) ever prescribed an SSRI had at least one prescription for citalopram, accounting for 64% of their prescriptions. Sertraline accounted for the majority of the remaining prescriptions (11% of the total for cases and 23% for controls).

Group I women who ever used citalopram while taking tamoxifen did not have a higher rate of breast cancer recurrence than women who never used citalopram while taking tamoxifen (Table 3; OR=0.79, 95% CI=0.40, 1.6). This OR was not substantially modified by duration of tamoxifen therapy (*P*=0.23 for test of homogeneity; data not shown). The approximately null effect persisted with adjustment for age category and ever/never use of another CYP2D6 inhibitor or SSRI (OR=0.85, 95% CI 0.42, 1.7). The effects were likewise approximately null within cumulative citalopram prescription categories (intermittent use OR=0.72, 95% CI 0.30, 1.7; regular use OR=1.1, 95% CI 0.37, 3.3). Citalopram use also had no substantial effect on recurrence in group II women (adjusted OR=0.78, 95% CI 0.17, 3.6), suggesting that citalopram does not directly affect the risk of breast cancer recurrence.

## Discussion

The results of this study do not support the hypothesis that citalopram, taken concurrently with tamoxifen, reduces tamoxifen's protective effect against breast cancer recurrence in early-stage patients whose tumour cells express the oestrogen receptor.

Our results extend the findings from an earlier study of 28 stage II and III breast cancer patients with recurrence and their matched controls at a single United States oncology centre, which also reported no substantial modification of tamoxifen effectiveness by concomitant use of SSRI inhibitors of CYP2D6 (Lehmann *et al*, 2004). These results may seem at odds with the strong biological rationale and *in vivo* evidence that support the hypothesis that CYP2D6 inhibition would reduce tamoxifen's prevention of breast cancer recurrence. It is possible, however, that SSRI medications could reduce the plasma concentration of tamoxifen's secondary metabolites without reducing its anti-tumorigenicity (Ponzone *et al*, 2004; Ratliff *et al*, 2004; Stearns *et al*, 2004). Tamoxifen doses as much as 20-fold lower than the typical US dose of 20 mg day^−1^ affect biomarkers of cardiovascular, bone, and tumour end points (Decensi *et al*, 1998, 2003), so the approximately three-fold reduction in the plasma concentration of tamoxifen's secondary metabolites associated with concomitant receipt of the SSRI paroxetine (Jin *et al*, 2005) may have little consequence.

The key mechanistic question may be whether reduced concentrations of active tamoxifen metabolites result in substantially reduced occupancy of the oestrogen receptor. Dowsett and Haynes (2003) estimated that, in postmenopausal women on a daily dose of 20 mg tamoxifen, tamoxifen and its metabolites occupy 9994 of 10 000 oestrogen receptors. Replicating their calculation using the plasma concentrations of tamoxifen and its metabolites in women with no *CYP2D6* variant allele (Jin *et al*, 2005), tamoxifen and its metabolites would occupy 9999 of 10 000 receptors in women not taking any SSRI and 9997 of 10 000 receptors in women taking the strong *CYP2D6*-inhibiting SSRI paroxetine. Steady-state concentrations of tamoxifen and its metabolites may be sufficient to manifest fully tamoxifen's antitumorigenic effect in postmenopausal women regardless of whether *CYP2D6* inhibition reduces the concentration of some tamoxifen metabolites.

Nonetheless, our results should be considered with the following limitations in mind. First, the majority of SSRI prescriptions in our study were for citalopram or its S-stereoisomer, both originally manufactured by Lundbeck, a company headquartered in Denmark. Citalopram is a modest inhibitor of *CYP2D6* compared with some other SSRI medications (Jeppesen *et al*, 1996). These more potent inhibitors may reduce tamoxifen's protection against breast cancer recurrence, but their interaction with tamoxifen would not have been well measured by this study.

Second, we have not collected genotype data to characterize functional *CYP2D6* variants (Hayhurst *et al*, 2001) that affect the metabolism of tamoxifen (Jin *et al*, 2005). The combination of genotype and receipt of *CYP2D6*-inhibiting medications has been related to tamoxifen effectiveness in a previous study (Goetz *et al*, 2007). We do not, however, expect ever-receipt of citalopram while taking tamoxifen to be related to *CYP2D6* genotype, as this genotype would be unknown to the patient and provider at the first citalopram prescription. This study's results therefore pertain to the usual clinical setting. In addition, *CYP2D6* genotype is unlikely to cause citalopram prescription, or to share a common causal ancestor, so *CYP2D6* genotype does not satisfy the requisite causal structure of a confounder (Greenland *et al*, 1999). It may be possible that *CYP2D6* genotype is related to adherence to citalopram prescription or to long-term maintenance of the prescription, resulting from differences in the occurrence of adverse drug reactions in women with the different alleles. Such a relation could confound the association between breast cancer recurrence and duration of citalopram prescription while taking tamoxifen. Some non-randomized studies suggest such a relation between genotype and SSRI adherence (Rau *et al*, 2004; Zourková *et al*, 2007), whereas others suggest no such relation (Stedman *et al*, 2002; Gerstenberg *et al*, 2003; Roberts *et al*, 2004; Hedenmalm *et al*, 2006; Sugai *et al*, 2006; Suzuki *et al*, 2006). In the only randomized trial, *CYP2D6* genotype was not related to either the occurrence of adverse events or to adherence to paroxetine prescription (Murphy *et al*, 2003). Paroxetine is the most potent *CYP2D6* inhibitor of tamoxifen metabolism among the SSRI class (Jin *et al*, 2005). If *CYP2D6* genotype does not affect receipt or adherence to SSRI prescription, then it cannot confound the association we have reported.

Last, we do not know the indications for which citalopram was prescribed to the study participants, although ordinarily it would be prescribed primarily to treat depression. SSRI may also be prescribed to treat hot flushes (Stearns, 2006), but such prescriptions are rare in Danish breast cancer patients.

Weighing against these limitations are the strengths of the data quality. This study relied upon the Danish Breast Cancer Cooperative Group's registry of breast cancer patients, which provides clinical trial quality data in a population-wide setting in the four Northern Danish Counties. For example, the positive predictive value of breast cancer recurrence recorded by the DBCG equaled 99.4% in a validation study (Hansen *et al*, 1997), showing that there are few false-positive recurrences registered in the DBCG. In addition, of 1888 local and distant recurrences identified by medical record review among 4455 breast cancer patients assigned to a DBCG protocol, 1813 (96%) were correctly registered as recurrences in the DBCG database, 74 (3.9%) were identified as breast cancer deaths, and only 1 (0.05%) was not identified as either a recurrence or breast cancer death.

The prescription databases are generated by a computerised pharmacy accounting system that sends data to the Danish National Health Service, which refunds part of the costs associated with prescribed drugs. Given the direct connection between receipt of prescription medications and the pharmacy accounting system of the Danish National Health Service, we expect the prescription records to have excellent validity. The prescriptions from the four counties are merged into a research database at Aarhus University. In Denmark, antidepressants are available only at pharmacies and the patient must have a prescription from a medical doctor. Therefore, the county prescription databases are expected to have high sensitivity and specificity for ascertainment of citalopram prescriptions in the source population. Furthermore, because the prescription records antecede the date of breast cancer recurrence, they are a prospective data source presumably immune to differential classification bias (Rothman *et al*, 2008).

Despite these advantages, the study yielded only 17 cases of breast cancer recurrence among tamoxifen-treated women who had used citalopram while taking tamoxifen. The study was designed with 80% power to detect an OR of 1.6, and ultimately had 90% power to detect an OR of 2.3.

The results presented herein are, nonetheless, important and timely. A United States Food and Drug Administration advisory committee recently recommended relabelling tamoxifen with information on gene–drug and drug–drug interactions mediated by *CYP2D6* (American Cancer Society, 2007). Furthermore, the current practice guidelines of the United States National Comprehensive Cancer Network note that some SSRI reduce the formation of active tamoxifen metabolites, that citalopram and venlaflaxine appear to have minimal impact on tamoxifen metabolism, and that ‘the clinical impact of these observations is not known’ (National Comprehensive Cancer Network, 2008). Breast cancer patients taking tamoxifen and their physicians may therefore be concerned about SSRI comedication, even when antidepressants are strongly indicated. Our results suggest that citalopram prescription does not reduce tamoxifen's prevention of breast cancer recurrence.

## Figures and Tables

**Table 1:** Frequency and proportion of cases of breast cancer recurrence and matched controls within group strata (I) expressing the oestrogen receptor and receiving at least 1 year of tamoxifen therapy (ERP+/TAM+), or (II) not expressing the oestrogen receptor, never receiving tamoxifen therapy, and surviving at least 1 year after diagnosis (ERP−/TAM−)

	**Group I: ERP+/ TAM+ (*n* (%))**		**Group II: ERP−/ TAM− (*n* (%))**	
	**Cases**	**Controls**	**Cases**	**Controls**
*Citalopram prescription*				
Ever	17 (9)	21 (11)	3 (3)	5 (6)
Never	167 (91)	163 (89)	84 (97)	82 (94)
				
*Other CYP2D6 inhibitors, including other SSRI*				
Ever	48 (26)	51 (28)	25 (29)	17 (20)
Never	136 (74)	133 (72)	62 (71)	70 (80)
				
*Diagnosis year* a				
1985–1993	33 (18)	34 (18)	13 (15)	11 (13)
1994–1996	32 (17)	29 (16)	17 (20)	18 (21)
1997–2001	119 (65)	121 (66)	57 (66)	58 (67)
				
*Age at diagnosis*				
35–44	13 (7)	11 (6)	15 (17)	12 (14)
45–55	38 (21)	34 (18)	37 (43)	29 (33)
55–65	91 (49)	93 (51)	26 (30)	29 (33)
65–70	42 (23)	46 (25)	9 (10)	17 (20)
				
*Menopausal status at diagnosis* a				
Premenopausal	24 (13)	24 (13)	30 (34)	30 (34)
Postmenopausal	160 (87)	160 (87)	57 (66)	57 (66)
				
*County of residence at diagnosis* a				
Aarhus	70 (38)	70 (38)	37 (43)	37 (43)
North Jutland	88 (48)	88 (48)	37 (43)	37 (43)
Viborg	15 (8)	15 (8)	9 (10)	9 (10)
Ringkøbing	11 (6)	11 (6)	4 (5)	4 (5)
				
*UICC tumour stage at diagnosis* a				
Stage I	7 (4)	7 (4)	4 (5)	4 (5)
Stage II	79 (43)	79 (43)	41 (47)	41 (47)
Stage III	98 (53)	98 (53)	42 (48)	42 (48)
				
*Histological grade*				
Grade I	31 (17)	33 (18)	4 (5)	17 (20)
Grade II	73 (40)	87 (47)	29 (33)	2 (2)
Grade III	44 (24)	24 (13)	38 (44)	22 (25)
Missing	36 (20)	40 (22)	16 (18)	46 (53)
				
*Surgery type*				
Breast conserving surgery	22 (12)	22 (12)	9 (10)	4 (5)
Mastectomy	162 (88)	162 (88)	78 (90)	83 (95)
				
*Radiation therapy*				
Yes	86 (47)	79 (43)	43 (49)	36 (41)
No	98 (53)	105 (57)	44 (51)	51 (59)
				
*Tamoxifen protocol*				
1 year	57 (31)	57 (31)	Not applicable	Not applicable
2 years	10 (5.4)	10 (5.4)		
5 years	117 (64)	117 (64)		
				
*Systemic adjuvant chemotherapy*				
Yes	21 (11)	24 (13)	70 (80)	61 (70)
No	163 (89)	160 (87)	17 (20)	26 (30)

a Variable included in risk set sampling to match controls to cases.

**Table 2:** Number of cases and controls receiving any prescription for each SSRI, and total number of prescriptions for each SSRI within group strata (I) expressing the oestrogen receptor and receiving at least 1 year of tamoxifen therapy (ERP+/TAM+), or (II) not expressing the oestrogen receptor, never receiving tamoxifen therapy, and surviving at least 1 year after diagnosis (ERP−/TAM−)

	**Group I: ERP+/TAM+** ***n* (no. of prescriptions) [range of no. per person]**		**Group II: ERP−/TAM−** ***n* (no. of prescriptions) [range of no. per person]**	
**SSRI name (ATC code)**	**Cases**	**Controls**	**Cases**	**Control**
Zimeldine (N06AB02)	0	0	0	0
Fluoxetine (N06AB03)	1 (4) [4–4]	4 (24) [1–13]	2 (12) [1–11]	0
Citalopram (N06AB04)^a^	16 (251) [1–53]	21 (123) [1–24]	3 (4) [1–2]	5 (64) [1–43]
Paroxetine (N06AB05)	2 (6) [1–5]	1 (1) [1–1]	1 (2) [2–2]	3 (20) [5–9]
Sertraline (N06AB06)	3 (32) [3–24]	7 (45) [1–15]	1 (2) [2–2]	1 (12) [12–12]
Alaproclate (N06AB07)	0	0	0	0
Fluvoxamine (N06AB08)	0	0	0	0
Etoperidone (N06AB09)	0	0	0	0
Escitalopram (N06AB10)^a^	1 (2) [2–2]	1 (3) [3–3]	0	0

a In the analysis, we defined citalopram exposure as any prescription for citalopram (N06AB04) or its S-stereoisomer escitalopram (N06AB10).

**Table 3:** Association between SSRI prescription and breast cancer recurrence within strata of (a) Group I, women with tumours that expressed the oestrogen receptor and who received at least 1 year of tamoxifen therapy (ERP+/TAM+) or (b) Group II, women with tumours that did not express the oestrogen receptor, who never received tamoxifen therapy, and who survived at least 1 year after diagnosis (ERP−/TAM−)

**Citalopram prescription**	**Cases/controls**	**Crude OR (95% CI)**	**Adjusted OR (95% CI)^a^**
*(a) Group I: ERP+/TAM+*			
Never user	167/163	1.0 (reference)	1.0 (reference)
Ever user	17/21	0.79 (0.40, 1.6)	0.85 (0.42, 1.7)
Intermittent use	10/14	0.69 (0.30,1.6)	0.72 (0.30, 1.7)
Regular use	7/7	0.97 (0.34, 2.8)	1.1 (0.37, 3.3)
			
*(b) Group II: ERP−/TAM−*			
Never user	84/82	1.0 (reference)	1.0 (reference)
Ever user	3/5	0.60 (0.14, 2.5)	0.78 (0.17, 3.6)

a Adjusted for age category and other CYP2D6-inhibiting medications (see the Supplementary online material for a complete list of these medications and the frequency of their use in the study population).
