# Supplementary material for: Tamoxifen's protection against breast cancer recurrence is not reduced by concurrent use of the SSRI citalopram
Source: Br J Cancer. 2008 Jul 29;99(4):616–21. doi: 10.1038/sj.bjc.6604533 (PMC2527838; doi:10.1038/sj.bjc.6604533)
Supplement: Supplementary online material [file 6604533x1.doc]

# Supplementary online material

Supplemental table of CYP2D6 inhibitors, substrates, and inducers used for adjustment of the association between breast cancer recurrence and ever/never use of citalopram.

| **ATC NAME** | **ATC CODE** | **ERP+/TAM+**  **cases/controls** | **ERP-/TAM-**  **cases/controls** |
| --- | --- | --- | --- |
| Histamine blocker |  |  |  |
| Cimetidine | A02BA01 | 9/9 | 3/4 |
| Ranitidine | A02BA02 | 0/2 | 2/0 |
| Antiemetic |  |  |  |
| Metoclopramide | A03FA01 | 12/5 | 12/1 |
| Ondansetron | A04AA01 | 2/0 | 0/0 |
| Antifungal |  |  |  |
| Terbinafine | D01BA02 | 0/2 | 0/1 |
| Antiarrythmia |  |  |  |
| Flecainid | C01BC04 | 1/0 | 0/0 |
| Amiodarone | C01BD01 | 0/0 | 0/1 |
| Beta blocker |  |  |  |
| Propranolol | C07AA05 | 5/0 | 2/1 |
| Metoprolol | C07AB02 | 10/0 | 3/4 |
| Timolol | S01ED01 | 3/0 | 0/0 |
| Antihypertensive |  |  |  |
| Carvedilol | C07AG02 | 1/0 | 1/0 |
| Non-steroidal antiinflammatory |  |  |  |
| Celecoxib | M01AH01 | 12/7 | 4/1 |
| Analgesic |  |  |  |
| Tramadol† | N02AX02 | 45/18 | 0/0 |
| Codeine†  Oxycodone† | R05DA04  N02AA05 | 12/9  12/0 | 0/0  0/0 |
| Antipsychotic |  |  |  |
| Chlorpromazin | N05AA01 | 0/0 | 0/2 |
| Levomepromazin | N05AA02 | 1/1 | 3/1 |
| Haloperidol | N05AD01 | 1/0 | 1/0 |
| Zuclopenthixol | N05AF05 | 1/1 | 2/2 |
| Perphenazine | N05AB03 | 0/1 | 2/0 |
| Risperidone | N05AX08 | 0/1 | 0/0 |
| TCA Antidepressants |  |  |  |
| Clomipramine | N06AA04 | 0/0 | 0/1 |
| Amitriptyline | N06AA09 | 3/7 | 5/3 |
| Nortriptyline | N06AA10 | 0/2 | 4/1 |
| Other Antidepressants |  |  |  |
| Moclobemid | N06AG02 | 1/0 | 0/0 |
| Mirtazapin | N06AX11 | 9/10 | 4/2 |
| Venlafaxin | N06AX16 | 6/2 | 0/1 |
| Opioids |  |  |  |
| Methadone† | N07BC02 | 0/1 | 0/0 |
| Cough Suppressants |  |  |  |
| Dexthromethorphan | R05DA09 | 0/1 | 0/0 |
| Steroid Hormone |  |  |  |
| Dexamethasone† | S01BA01 | 2/1 | 0/0 |

†Not included in the adjustment for ever/never use of a CPY2D6 inhibitor or substrate because the drug may be used to treat breast cancer recurrence or its symptoms.
